# A Protocol for the Generation of Treatment-naïve Biopsy-derived Diffuse Intrinsic Pontine Glioma and Diffuse Midline Glioma Models

**DOI:** 10.33696//Neurol.1.025

**Published:** 2020-12

**Authors:** Matt C. Biery, Alyssa Noll, Carrie Myers, Shelli M. Morris, Conrad A. Winter, Fiona Pakiam, Bonnie L. Cole, Samuel R. Browd, James M. Olson, Nicholas A. Vitanza

**Affiliations:** 1 Fred Hutchinson Cancer Research Center, Seattle, WA, USA; 2 Molecular and Cellular Biology Graduate Program and Medical Scientist Training Program, University of Washington, Seattle, WA, USA; 3 Department of Laboratories, Seattle Children’s Hospital, Seattle, WA, USA; 4 Department of Laboratory Medicine and Pathology, University of Washington School of Medicine, Seattle, WA, USA; 5 Division of Neurosurgery, Department of Neurological Surgery, University of Washington, Seattle Children’s Hospital, Seattle, WA, USA; 6 Division of Hematology/Oncology, Department of Pediatrics, Seattle Children’s Hospital, University of Washington, Seattle, WA, USA

## Abstract

Diffuse intrinsic pontine glioma (DIPG) is a universally fatal tumor of the brainstem, most commonly affecting young children. Due to its location, surgical resection is not achievable, but consideration of a biopsy has become standard practice at children’s hospitals with the appropriate neurosurgical expertise. While the decision to obtain a biopsy should be directed by the presence of atypical radiographic features that call the diagnosis of DIPG into question or the requirement of biopsy tissue for clinical trial enrollment, once this precious tissue is available its use for research should be considered. The majority of DIPG and diffuse midline glioma, H3 K27M-mutant (DMG) models are autopsy-derived or genetically-engineered, each of which has limitations for translational studies, so the use of biopsy tissue for laboratory model development provides an opportunity to create unique model systems. Here, we present a detailed laboratory protocol for the generation of treatment-naïve biopsy-derived DIPG/DMG models.

## Introduction

Diffuse intrinsic pontine glioma (DIPG) is an aggressive brain tumor that arises in the ventral pons during middle childhood. Approximately 300 children are diagnosed with DIPG annually in the United States, making DIPG one of the most common malignant tumors of childhood [1]. Due to its diffuse growth through critical brainstem structures, DIPG is unresectable. Clinical trials of radiation-sensitizing agents, intensive chemotherapies, maintenance chemotherapies, and targeted agents have yet to make a significant impact though some conventional chemotherapy regimens may modestly improve and extend life [2,3]. Therefore, the standard of care is limited to focal radiation, most commonly to ~54 Gy, which often improves symptoms and extends life by ~3 months [4]. Ultimately, with an average overall survival ~11 months, DIPG is the leading cause of pediatric central nervous system (CNS) tumor death [5]. Considering the unresectable pattern of growth and lack of long-term survivors, DIPG is often diagnosed by imaging alone. While biopsy is feasible, it must only be performed by experienced neurosurgeons because transient, though sometimes permanent, neurologic deficits can result [6]. This constellation resulted in decades of extremely limited DIPG tissue for study.

Over the past decade, the development of patient-derived DIPG models has begun to shed light on DIPG pathophysiology. Several rapid autopsy protocols have been published that describe the process of using post-mortem tissue to create cell cultures that can be used for *in vitro* testing and used for the generation of orthotopic xenograft models [7-9]. These efforts in combination with larger collaborative efforts within the field to share models between investigators led to significant progress in understanding the biological mechanisms underlying DIPG and identified several novel therapies ranging from epigenetically-targeted drugs (NCT02717455) to chimeric antigen receptor (CAR) T cell trials (BrainChild-03, NCT04185038) that are currently being evaluated in clinical trials [10,11]. A series of notable whole-genome sequencing studies identified a recurrent *K27M* mutation in the genes encoding histone 3 in 85% of DIPG tumor samples - the first demonstration of a histone mutation driving oncogenesis [12-14]. Subsequent research associated this oncohistone with other diffusely infiltrative childhood CNS tumors, such as thalamic and spinal high-grade gliomas [15,16]. Therefore, in 2016 the World Health Organization (WHO) created a distinct diagnostic category: H3 K27M-mutant diffuse midline glioma (DMG) [17]. While patient-derived cell cultures and xenograft models generated by rapid autopsy protocols are generously donated and have been instrumental to the field’s progress, they are associated with several limitations. Successful generation depends on sample viability upon receipt, which is influenced by the post-mortem interval to autopsy as well as time required for tissue transportation. An important consideration when using these models is their history of exposure to radiation, often in addition to various cytotoxic, molecularly-targeted, or even epigenetically-targeted therapies. Therefore, the biology of models derived from post-mortem tissue may not reflect the tumor biology present at the time of diagnosis or progression in the patient from whom it was derived. This is exemplified by studies demonstrating differences in mutation rates between post-mortem samples and biopsy specimens. Untreated biopsy samples had a lower average mutation rate compared to autopsy samples (mean of 0.76 vs 1.2 mutations per megabase), suggesting an accumulation of mutations over the course of disease progression [18]. Several genomic alterations are more predominant in autopsy samples, such as PDGFRA gains/amplifications which are detected twice as frequently in autopsy samples [19]. Ultimately, these features of autopsy-derived models may influence clinical translatability.

The discovery of *H3 K27M* mutations spurred efforts to create genetically-engineered mouse (GEM) models of DMG. Unlike conventional patient-derived xenograft models, GEM models are created in the context of a relevant microenvironment within an immunocompetent host, exploiting viral transduction or *in utero* electroporation technologies to manipulate genomes of cells in distinct brain regions at various mouse ages. The ability to direct genetic alterations at key stages of brain development makes GEM models extremely useful for studying DMGs. While they have enormous preclinical potential, these models are not without caveats. One mouse model utilizes the RCAS viral system to promote over-expression of *PDGFB* in brainstem neural progenitor cells in combination with *Ink4a-ARF* and *TP53* loss [20]. This drives the formation of aggressive murine brainstem gliomas that share histopathological similarities with DIPG. However, *PDGFB* overexpression or amplification is uncommon in pediatric gliomas [21] and an analysis of *PDGFB*-driven brainstem glioma models demonstrated that they are characterized by a pro-angiogenic phenotype with increased expression of proneural genes more reflective of adult glioblastomas [22]. A growing number of models also utilize *in utero* electroporation to transfect brainstem neural progenitor cells with *DNp53* and *H3.3 K27M* in combination with various molecular drivers to create faithful murine models of DMG [22,23]. While this system allows researchers to investigate the role of specific genetic drivers, like any GEM model, it represents only a subset of the disease spectrum.

Although brainstem biopsies historically have been considered routine in some parts of the world, surgical biopsies of DIPG were discouraged for many years in the United States. However, several reports support the safety and feasibility of neurosurgical biopsy [6,24,25]. Beyond confirmation of clinical diagnosis, collection and genetic characterization of biopsies can satisfy enrollment criterion for a growing number of molecularly- and immunologically-targeted DMG clinical trials. Furthermore, as we detail here, biopsy samples can provide sufficient tissue to generate laboratory models while also providing enough tissue for histopathologic diagnosis, clinical sequencing, and tissue sharing amongst consortia. A multi-center retrospective analysis found that, compared to autopsy samples, biopsy-derived tissue more reliably produced a sustainable cell culture (84.2% vs. 38.2%), while primary cultures generated from post-mortem samples were more likely to engraft in mice (86.7% vs 47.4%) [26]. As performing diagnostic biopsies for DIPG inches toward becoming standard of care, there will be increased opportunities to generate patient-derived treatment-naïve models. Here, we present a protocol to generate preclinical models from biopsy-derived DMG specimens with the aim to spur efforts to develop more translationally relevant models. We show that our creation of these model systems is feasible both as *in vitro* cultures and xenograft *in vivo* models. Ultimately, we hope these methods will be reproduced to help build an international stable of shared treatment-naïve patient-derived DIPG/DMG models to serve as a compliment to existing systems in a collaborative effort to better understand currently available therapies, to inform the best next-generation combinatorial clinical trials, and to promote discovery of new treatment options for children with this devastating disease.

## Materials and Methods

### Biopsy acquisition and histopathology

The Mayfield cranial stabilization and fixation system manufactured by Integra (Cincinnati, OH) is used to position the pediatric patient for surgical biopsy. The Stealth Vertek biopsy device, manufactured by Medtronic (Minneapolis, MN) is employed to collect specimens. Eight cores are typically acquired, as described in the Results section. Each needle core biopsy collected for cell culture and direct orthotopic implants is stored in RPMI (containing 500 mL RPMI-1640 with L-glutamine, and no phenol red (ThermoFisher cat.# 11835-030), 50 mL aliquot fetal calf serum (HyClone SH30070.03), 5 mL penicillin-streptomycin solution (ThermoFisher 15140-122), and 5 mL gentamicin (Sigma G1272)) at 4°C until ready for continued processing. Standard hematoxylin and eosin (H&E) staining is performed on primary biopsy tissue to assess tumor cell morphology. Staining for the diagnostic H3 K27M protein is performed with a 1:1200 antibody dilution (EMD Millipore cat.# ABE419) using the iView DAB detection kit (Roche cat. #760-091) on the Ventana Benchmark Ultra automated platform.

### Cell culture and medium

Neurocult™ NS-A human proliferation kit (StemCell Technologies cat.# 05751) is used as the basal medium. Medium additions include: 1x Glutamax (ThermoFisher cat.# 35050079), 40 ng/mL EGF (Peprotech cat.# AF-100-15), 40 ng/mL FGF basic (Peprotech cat. # 100-18b) to generate Neurocult Complete (NC). For the first month of culture growth, NC medium is further supplemented with 1x Antibiotic/Antimycotic (ThermoFisher cat.# 15240062) to control potential microbial contamination. Where described, plasticware is coated overnight with laminin derived from Engelbreth-Holm-Swarm sarcoma tumor basement membranes (Sigma-Aldrich cat.# L2020) at a 1:100 or 1:50 dilution in DPBS (ThermoFisher cat.# 14190144) in a 37°C incubator. Accutase (Innovative Cell Technologies cat.# AT104), a gentle enzyme mixture with proteolytic and collagenolytic activity, is used to liberate adherent cells from plastic and dissociate durable cell clumps. Cells are maintained in vented tissue culture flasks at 37°C/5% CO_2_ with incubator humidification.

### Lentiviral construction and transduction

To generate the pLenti CMV mCherry-T2A-Luc blast plasmid, the mCherry-T2A-Luciferase reporter DNA was PCR amplified from the NL4-3 mCherry Luciferase plasmid (a gift from Warner Greene, Addgene plasmid #44965; http://n2t.net/addgene:44965; RRID:Addgene_44965) and Gibson cloned downstream of the CMV promoter in AgeI and SalI digested pLenti CMV GFP Blast (659-1) (a gift from Eric Campeau and Paul Kaufman, Addgene plasmid #17445; http://n2t.net/addgene:17445; RRID:Addgene_17445) [27,28]. Following production of virus in HEK 293T cells, 3 mL of virus containing supernatant is added to a 20 mL cell culture in a vented T75 flask at 1:20 dilution and cells are allowed to develop integrations for 4 days prior to selection with blasticidin at 1 ug/mL for an additional 3 days to enrich transduced cells with stable integration.

### *In vivo* orthotopic implantation and histopathology

NOD. Cg-Prkdcscid Il2rgtm1Wjl/SzJ (NSG) mice are bred in house and aged 6-8 weeks. Animals are housed according to standard guidelines with 12-hour light-dark cycles. All procedures are approved by international Animal Care and Use Committee (IACUC). Tumor tissue is gently triturated into small fragments, or cells from tissue culture are suspended in NC medium at approximately 100,000 cells per 2 μL injection volume. To initiate intracranial growth of tumor models, a 0.9 mm burr hole is made at 2 mm lateral, 1 mm caudal of lambda, and a pipette tip is used to deliver cells directly under the dura. If cells are transduced with luciferase, mice are imaged regularly by IVIS Spectrum to monitor tumor growth. Otherwise, signs of acute tumor development include cranial bulge, head tilt, loss of balance, loss of weight, or hunched posture.

Histologic sections of formalin fixed paraffin embedded orthotopic mouse brain tumors were cut at 6 microns. H&E staining was performed by hand using Harris hematoxylin and alcoholic Eosin Y. Immunostaining for albumin and Ki-67 was performed on the Ventana Discovery Ultra platform, using the ChromoMap DAB detection kit (Roche cat. # 760-159) with anti-Rabbit HQ (Roche cat. #760-4815) and anti-HQ HRP (Roche cat. #760-4820). The albumin antibody (GeneTex cat. #GTX102419) was used at a 1:1500 dilution. The Ki-67 antibody (Dako cat. #M7240 was used at a 1:100 dilution followed by a rabbit anti-mouse secondary antibody at 1:200 (Abcam cat. #ab133469).

## Results

### Biopsy acquisition

**Figure 1:**
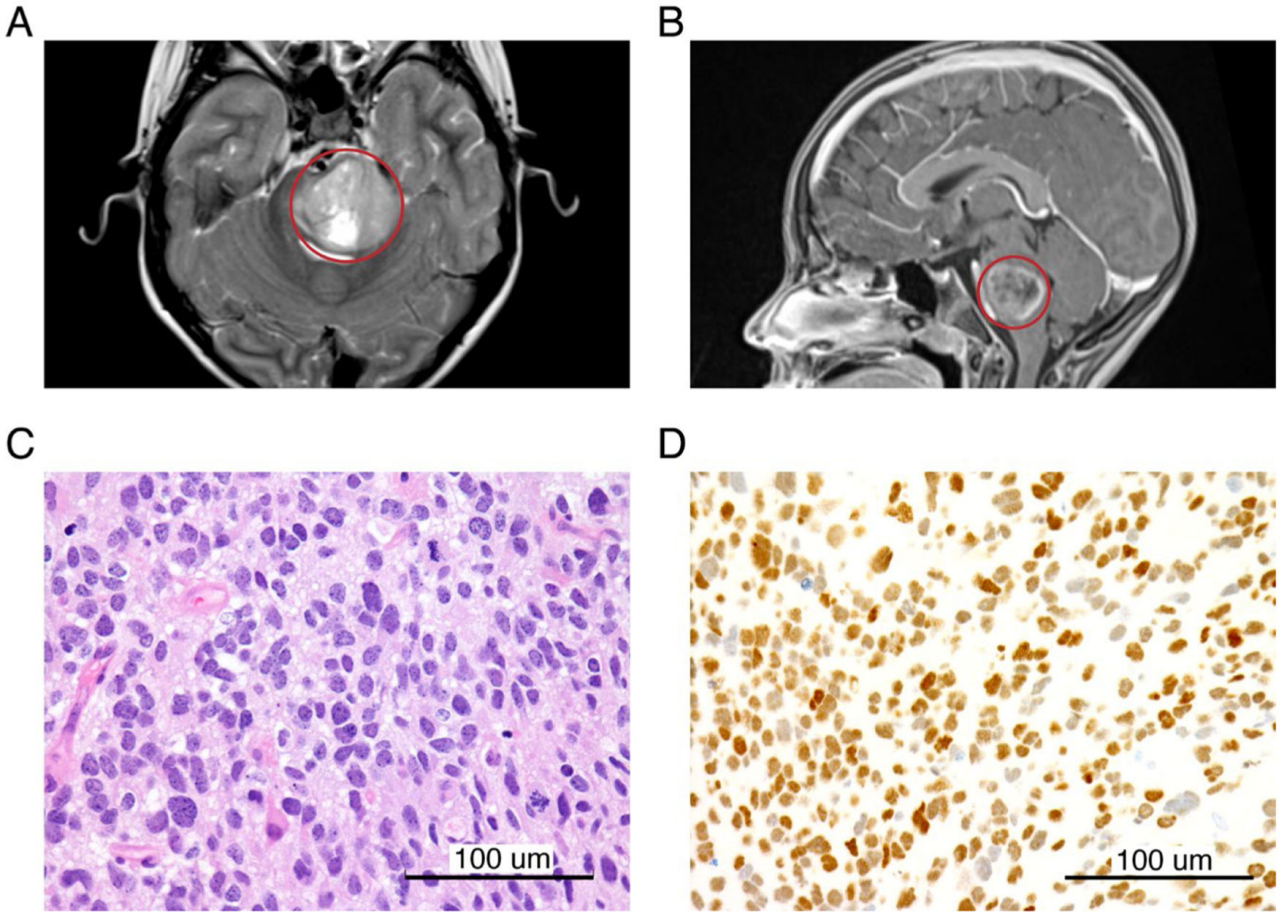
Radiographic and histopathologic findings of DIPG. Magnetic resonance images including representative **(A)** axial T2 and **(B)** sagittal T1 post-contrast images (tumor is circled in red). **(C)** H&E and **(D)** H3 K27M immunohistochemistry (scale bar = 100 μm).

While pontine DMG are often described as having a “classic” imaging appearance, there are inconsistencies in imaging interpretations [29]. However, common MRI features include diffuse hyperintensity throughout the majority of the ventral pons on T2 imaging with a lack of enhancement on post-contrast T1 imaging, as present in the diagnostic imaging of our most recent DMG model (PBT-29FH; Figures 1A and 1B). While standard initial therapy is focal radiation to ~54 Gy and some studies do not require histopathologic confirmation, early biopsy collection presents an opportunity to evaluate for potentially targetable mutations and often offers patients a broader range of early phase clinical trials [4]. As biopsies also provide valuable treatment-naïve tumor tissue, we developed an operating room-to-laboratory workflow to maximize research tissue utilization and attempt to develop treatment-naïve DIPG/DMG models [30].

At Seattle Children’s Hospital and the University of Washington (UW) Harborview Medical Center, the patient or caregiver is consented by the neurosurgeon for the surgical needle core biopsy and independently is offered to consent for tissue donation for research (Seattle Children’s Institutional Review Board approval #14449). Stereotactic biopsies are performed via a posterior fossa trans-middle cerebellar peduncle approach to the brainstem [25]. Once intubated and positioned prone in rigid, Mayfield fixation, we utilize a small linear incision off-midline and create a burrhole. Once the dura is opened, we use the Medtronic Stealth Vertek biopsy device to obtain the samples. Using the MRI preoperative imaging, we determine the best biopsy location, typically this is closest to the lateral, posterior edge of the tumor, also areas that might diffusion restrict or have more abnormal features are preferentially targeted if felt to be safe. We attempt to obtain 8 core specimens, 4 at the first depth and second group of 4 specimens at a slightly deeper or more superficial location along the same trajectory. Samples are taken circumferentially at the target depth (i.e. at 12, 3, 6, and 9 o’clock locations). The specimens are placed on wet-telfa and sent to the clinical laboratory. The incision is closed, a post-operative CT scan is obtained, and the patient is observed overnight in the PICU before commonly being discharged on post-operative day 1. The risk of non-diagnostic specimen is uncommon and the risk of adverse events is generally low but includes hemorrhage, cranial nerve palsies, focal neurologic deficits, and worsening edema [6,25,31].

### Pathologic specimen handling and evaluations

As frozen section results will not alter the surgery, intra-operative frozen sections are not performed as part of this protocol. Foregoing a frozen section also allows conservation of precious tumor tissue for other purposes. In a typical surgery eight stereotactic tissue cores are sent to histology in two separate specimen containers (four cores per container). Two cores from each container are submitted for routine formalin fixation and paraffin embedding; this amount of tissue has been found to be sufficient for most of the routine histologic and molecular profiling studies needed for patient care. Three cores are frozen and saved for either research or clinical use if needed. And finally, one core is submitted in RPMI for model creation.

While laboratory distribution of tissue is very valuable, the primary goal of any attained biopsy must be appropriate diagnosis with tissue available for histopathology and molecular testing. Figure 1 captures histopathologic and molecular features from the diagnostic biopsy sample of the patient from whom PBT-29FH was derived. Tumor samples are subjected to a standard staining panel to characterize the tumor that includes H&E and H3 K27M staining (Figures 1C and 1D). The H3 antibody is specific for the mutated form of histone 3, both the protein isoforms encoded by *H3F3A* (H3.3) and *HIST1H3C* (H3.1) genes. After the histologic diagnosis has been made, additional FFPE scrolls are submitted to the UW genomics laboratory for total nucleic acid extraction followed by targeted DNA sequencing via UW-OncoPlex [32].

### Initiation of tissue culture

**Figure 2:**
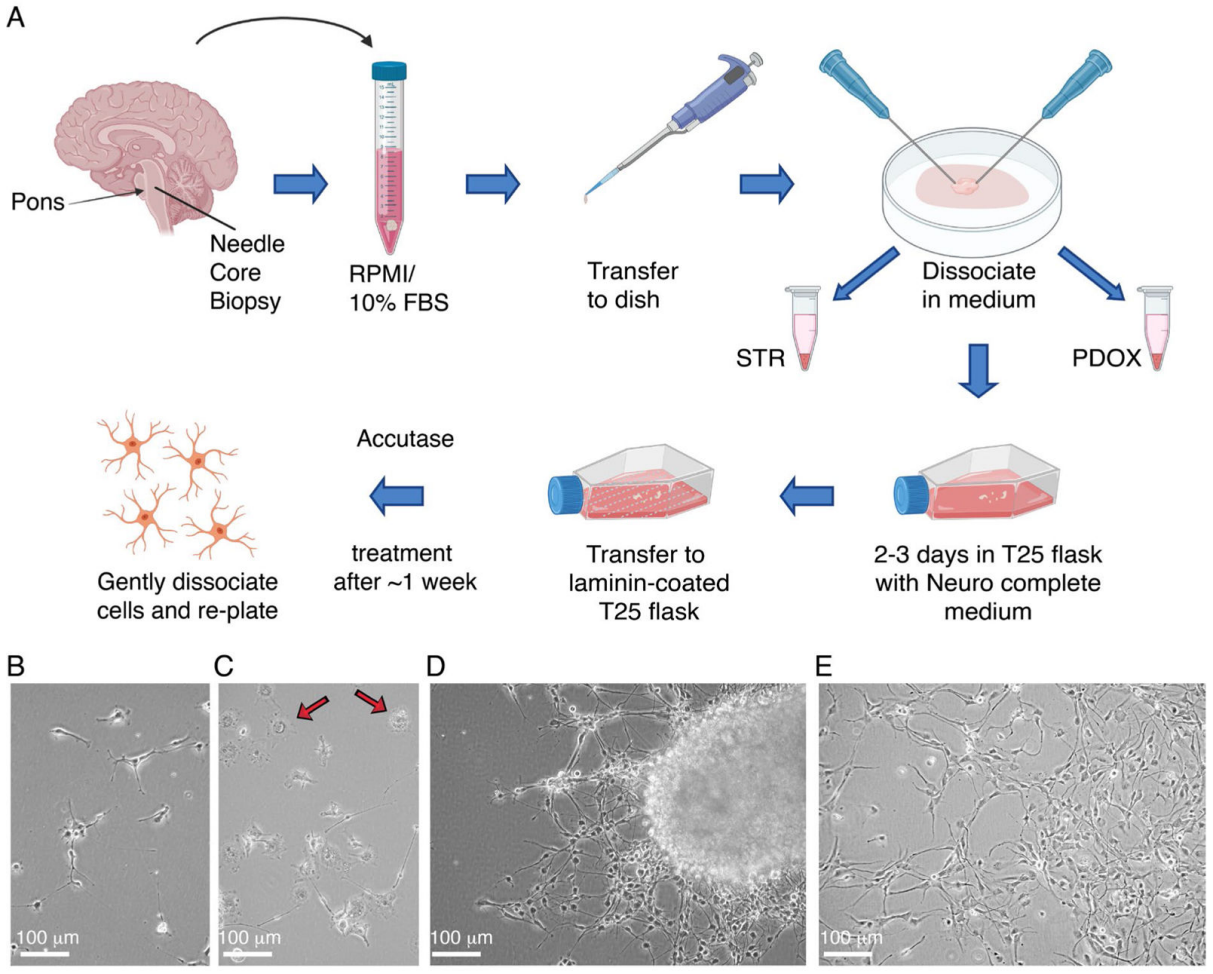
Establishment of tissue culture models from biopsy. **(A)** Diagram of general workflow for the establishment of tissue culture models from biopsy. Representative images of **(B)** early adherent tumor cells, **(C)** macrophages (indicated by arrows), **(D)** migratory tumor cells, and **(E)** tumor cell monolayer from tissue culture establishment of PBT-29FH.

Biopsy specimens are received on wet ice in the research laboratory, generally within 4 hours of surgical acquisition. A typical specimen has sufficient material to initiate a tissue culture, implant orthotopic xenografts in immunodeficient mice, and supply material from which gDNA can be extracted for STR analysis. The overall flow of the process is diagrammed in Figure 2A. A 1 mL micropipettor is used to retrieve the specimen from the collection tube and carefully transfer to a 0.5 mL puddle of Neurocult complete (NC, see Materials and Methods) centered in a sterile 10 cm dish, a convenient size for mechanical manipulation. Two 18-gauge needles are then used to tease the tissue fragment apart and expose additional surface area, one needle to immobilize the specimen against the surface and a second to pull tissue away from the anchor point. A small fragment is set aside to snap freeze and store for a downstream gDNA isolation that will be used for the STR profiling comparison of initial tumor to generate culture and/or xenografts. The remainder of the sample is then split approximately into two halves, one for the TC line attempt and the other for orthotopic implants in mice. The portion to be used for implants is set aside in a small volume of NC in a microfuge tube on ice until the time of implant surgery. Establishing and passaging tumor tissues as patient-derived orthotopic xenograft (PDOX) models in mice has been described previously [33].

Glioma cells are inherently fragile *ex vivo*, so any steps that can be taken to minimize extensive mechanical stress are beneficial. The sample has already been partially dissociated as described above, and no more physical manipulation is required prior to the initial incubation in culture. The next step is to simply transfer the entirety of the cell suspension, small chunks and remnants from the 10 cm dish to a vented T25 flask containing 10 mL of NC, supplemented with 1X Antibiotic/Antimycotic. The dish may also be rinsed with an additional 2-3 mL of NC if there is evidence of remaining tissue, and suspension combined with the tissue in the flask. Samples are incubated for at least 2-3 days at 37°C/5% CO_2_. The purpose of this incubation step is to allow the tumor cells to become acclimated to the medium, all the while residing in the proximity of cells and stroma present within the original tumor. Often, new growth can be detected after a few days by visual inspection, as peripheral outgrowths on tissue fragments. When scanning a range of focal planes when inspecting tumor fragments, viable cells on the perimeter will often have visible dendrites extending from their surface, appearing like small black hairs. Visual inspection of the whole flask will likely reveal the following:

- Sporadic adherent cells with glial morphology on the plastic
- Large vacuolized macrophage adhering strongly to the plastic with an appearance like fried eggs
- Red blood cells (RBCs) throughout the culture, and variable concentrations in clots in the tissue fragments
- Vasculature, predominantly capillaries, characterized by presence of RBCs throughout lumens of the vessels
- Possible fibroblastic cells
- Differentiated normal CNS cells of mixed morphologies

Success with a diverse repertoire of tissue culture lines derived from pediatric CNS tumor patients has in part been realized by pretreatment of the flask and dish surfaces with laminin derived from Engelbreth-Holm-Swarm sarcoma tumor basement membranes. While not all cell cultures require the laminin for growth, our laboratory has observed improved success when culturing atypical teratoid rhabdoid tumors (ATRT) and high-grade glioma (HGG) cultures on laminin-coated surfaces. Additionally, while many autopsy-derived DIPG lines grow relatively well as spheroids [8,34-36], some of our treatment-naïve isolates have fared poorly in this initial mode of growth. The first treatment-naïve DIPG line we successfully expanded in culture, PBT-09FH, demonstrates a propensity to form spheroids with dark, granular cores [30]. Upon attempted passaging of these spheroid cultures, cell viabilities are low, and debris is abundant. Considerable improvement is observed when plasticware treated with a 1/50^th^ dilution of laminin in PBS buffer overnight in a tissue culture incubator is utilized. For most DIPG lines attempted, a 1/100^th^ dilution of laminin has been sufficient. Doubling the concentration has proved to be beneficial to promote adherence for challenging isolates. Since the cells tend toward growth as a monolayer on laminin, less mechanical insult occurs in the process of breaking cells apart during downstream passaging.

Over time, many cells in the culture will adhere to the plastic and begin to stretch out dendrites from the cell bodies to form distant attachments from the core body (Figure 2B). Cell bodies themselves will also elongate as they spend time on laminin matrix. Macrophages, likely evident in the initial plating as adherent cells, will flatten out with abundant vacuoles in their centers (Figure 2C). Red blood cells are visible at variable densities depending on the specimen, starting out as translucent readily identifiable bodies, and degenerating to opaque, granular debris over time. This natural degradation of this cell type negates the need to purposefully lyse the cells early in the culture and expose the glioma cells to unnecessary insult. Larger pieces of tissue will often adhere to the coated surface and seed a sort of colony, with tumor cells migrating radially away from the base of the attachment (Figure 2D). This migration serves to separate viable cells from stroma and cellular debris present in the tumor sample. Surprisingly high proportions of the tissue chunk can be composed of viable cells in some cases. Nonviable material will accumulate in the center of the forming colony as the viable mobile cells evacuate the tissue.

Depending on the density of tumor cells within the particular biopsy, visible expansion of tumor cells can be observed in ~1-4 weeks. Expansion to a laminin-coated T75 flask is appropriate when the cells achieve approximately 75% confluence. Media within the flasks is renewed twice weekly. Accutase is employed at this stage to enhance dissociation of cells from the tumor pieces. The suspension component is collected by centrifugation at ~300 G for 4 minutes, aspirated and combined with the cells liberated from the flask plastic in Accutase. The combined cells are then incubated an additional 10 minutes at room temperature, before being pelleted again, and resuspended in fresh NC. Although the number of cells in a monolayer may be visibly increasing, larger chunks are retained alongside monodispersed cells to maximize potential for more deposition of cells in the monolayer as the culture progresses. All subsequent expansions involve disassociation of adherent cells from the flask using Accutase prior to re-plating. Due to much stronger adherence, macrophages are readily left behind on the plastic with successive passages. In contrast, tumor cells release from plastic rapidly with Accutase treatment. Eventually, a more evenly distributed monolayer evolves (Figure 2E). The progression of surface area advances from a single T25 to a single T75, to two T75S, to one T225. Once a dense T225 flask of cells is reached, cells are harvested, counted and stored in NC/10% DMSO at cell densities ranging from a minimum of one million cells/mL to 5 million cells/mL. Cells are also seeded for continued expansion, and storage at subsequent passages.

### Labeling of cells via lentiviral transduction

Once a tissue culture line has been successfully established from a patient tumor, it is often advantageous to introduce fluorescent protein labels like mCherry Red or GFP and/or luciferase. These labels can be useful for not only monitoring the cells during real-time cell-based assays, but also to provide reliable tags for IHC staining of brain/tumor resections from animals. Luciferase-bearing cell cultures have proven to be a very useful for monitoring progress of orthotopic tumor development via *in situ* luminescence imaging. Thus, a lentiviral insert encoding a fluorescent protein (mCherry), luciferase, and a selectable drug resistance promotes broad functionality for downstream studies (lentiviral expression construct diagram and cells transduced with this construct, Supplemental Figure 1).

### Engraftment of patient-derived cells in mice

**Figure 3:**
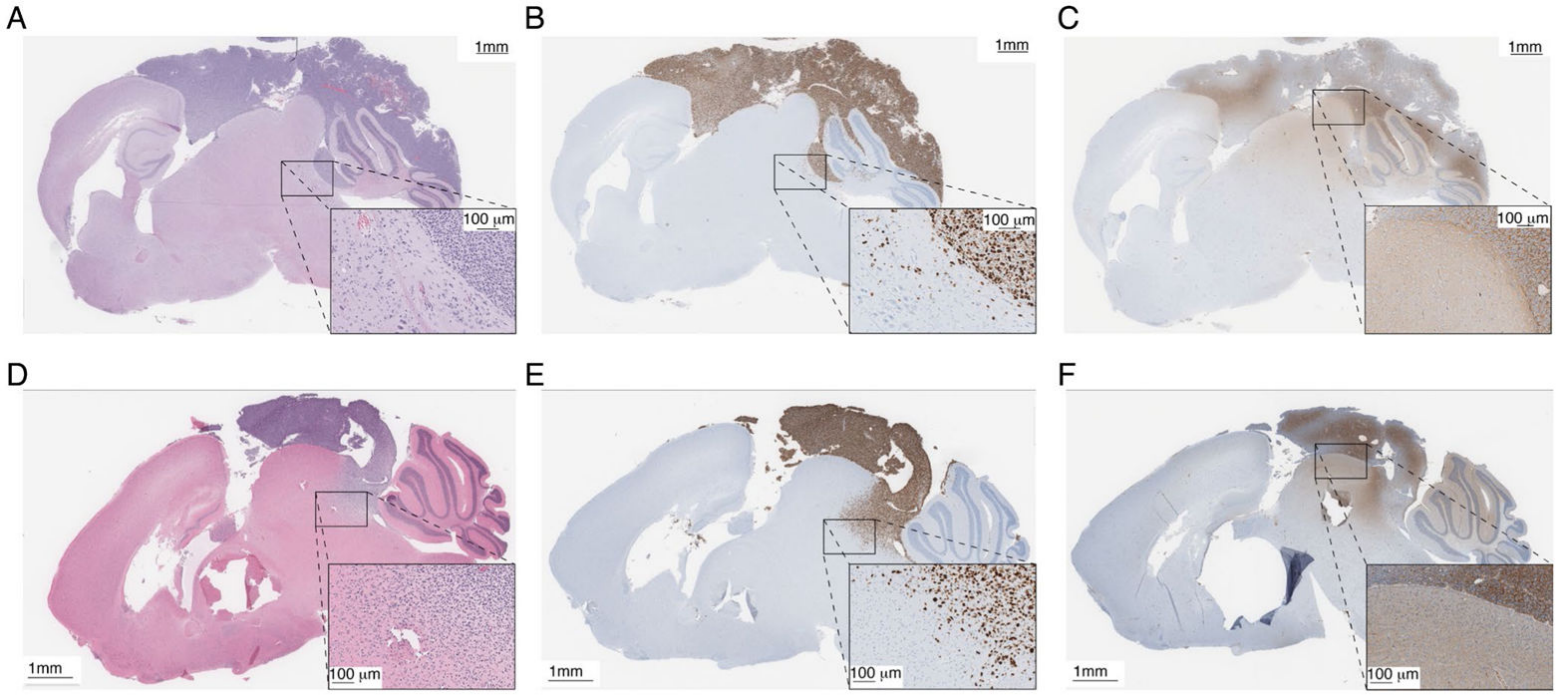
Orthotopic xenograft models. Immunohistochemistry of implanted tissue culture cells, including **(A)** H&E, **(B)** Ki67, and **(C)** albumin. Immunohistochemistry of implanted primary tumor including **(D)** H&E, **(E)** Ki67, and **(F)** albumin.

Orthotopic xenografts are attempted in parallel to an tissue culture attempts. An example of a cerebellar implant originating from 200,000 PBT-29FH tissue culture cells at passage five and a cerebellar implant originating directly from the PBT-29FH biopsy tissue are compared histologically in Figure 3. Grossly, the two tumors are strikingly similar, forming prominent dorsal masses on the brain and initiation of diffuse infiltration into the cerebellum and midbrain. Each developed in ~7 weeks (Figure 3A and 3D). The tumor cells are identified by strong staining with human specific Ki67 antibody (Figure 3B and 3E), while albumin staining (Figure 3C and 3F) is performed to characterize blood-brain-barrier disruption. This particular model demonstrates significant disruption of the blood-brain-barrier, as indicated by strong extracellular albumin deposition in the focal tumors, and diffuse extracellular staining in the mouse brain tissue at the boundaries of the tumor and in regions diffusely infiltrated by tumor cells. Successful establishment of the cells in culture, and subsequent success of implanting cultured cells thus provides a valuable starting platform from which to genetically engineer the tumor cells for more complex interrogation of the disease *in vivo*.

## Discussion

Despite ongoing efforts to improve outcomes, the prognosis of patients with DMG remains dismal. International efforts to create and distribute faithful patient-derived DIPG and DMG models, predominantly obtained from autopsy-derived tissue samples, has led to an improved understanding of the molecular and genetic drivers of this disease. This knowledge, in combination with demonstrated safety of the procedure, spurred an increase in the use of stereotactic biopsy to confirm a clinical diagnosis of DMG and to guide selection of molecularly targeted treatments. The growing availability of diagnostic tissue samples creates new opportunities to generate treatment naïve patient-derived models for research use. Here, we describe a protocol for the processing of DMG biopsy tissue cores to reliably develop cell culture lines that can ultimately be used for *in vitro* and *in vivo* preclinical testing alongside autopsy-derived DMG cell cultures. These complimentary preclinical models, reflecting the original tumor biology at different points of disease progression, will be invaluable for investigating DMG pathophysiology and identifying novel therapeutic strategies.

Our protocol takes several steps to optimize tumor cell viability and increase the likelihood of generating a successful DMG cell culture line. As DMG tissue is extremely delicate, we aim to minimize cellular stress at every point in the protocol. For example, we intentionally minimize mechanical and enzymatic dissociation steps by simply teasing apart the tissue carefully with two 18-gauge needles before transferring the tissue fragments to a laminin-coated tissue culture flask. The use of laminin allows the tumor cells to grow out as a monolayer, improving the diffusion of growth factors to cells and reducing the hypoxia that often occurs in the center of developing neurospheres when DMG cells are grown in suspension. Other steps taken in this protocol to improve sample viability include the decision to forgo lysis of red blood cells, in order to avoid placing unnecessary osmotic stress on tumor cells. It is important to note that the time required to generate a confluent T25 flask varies between samples and may take anywhere from 2-4 weeks, so patience will serve the researcher well.

There are inherent challenges in this process of generating a cell culture from a biopsy. One limitation of this protocol is the small amount of tissue provided by needle core biopsies. Given the diffuse nature of DMG, it is possible that the tissue cores may contain only sporadic tumor cells. Additionally, the logistics of obtaining diagnostic tissue specimens via sensitive surgery are challenging. While DIPG biopsy is becoming more standard and risks of Grade 3 and 4 toxicities is low, neurologic adverse events are common and can be permanent. Therefore, it is critical that only experienced neurosurgeons perform the biopsies following thoughtful discussions with family that highlight the central role played by the detailed histopathologic and molecular analysis of samples in diagnosis, treatment, or clinical trial enrollment. We advocate that samples are only allocated for laboratory research following confirmation of adequate diagnostic tissue and that appropriate consent is obtained, which should include a discussion of the emotional and psychologic implications of having a child’s tumor growing in a lab and the potential disappointment of not being able to generate a culture. Finally, such precious tissue should only be obtained and processed after thoughtful exploration of an institution-specific protocol that supports success.

Ultimately, the pediatric CNS tumor community has undergone an explosion of available models, though due to critical differences they should be thoughtfully selected depending on the experimental design and aims. This protocol offers a feasible method to create biopsy-derived treatment-naïve models that could fill a critical role in basic and translational studies. It could also be modified to generate patient-derived cell cultures other CNS tumors when only biopsy is achievable. By sharing our protocol for the unique generation of treatment-naïve biopsy-derived DMG models, we hope to drive an increase in developed models that can be used to deepen our understanding of this devastating disease and to identify promising therapeutic strategies to improve the lives of affected children.

## Supplementary Material

Supplementary Figure 1
