# Supplementary figures and images for: A Protocol for the Generation of Treatment-naïve Biopsy-derived Diffuse Intrinsic Pontine Glioma and Diffuse Midline Glioma Models
Source: J Exp Neurol. Author manuscript; Available in PMC 2021 Mar 24. (PMC7990285; doi:10.33696//Neurol.1.025)

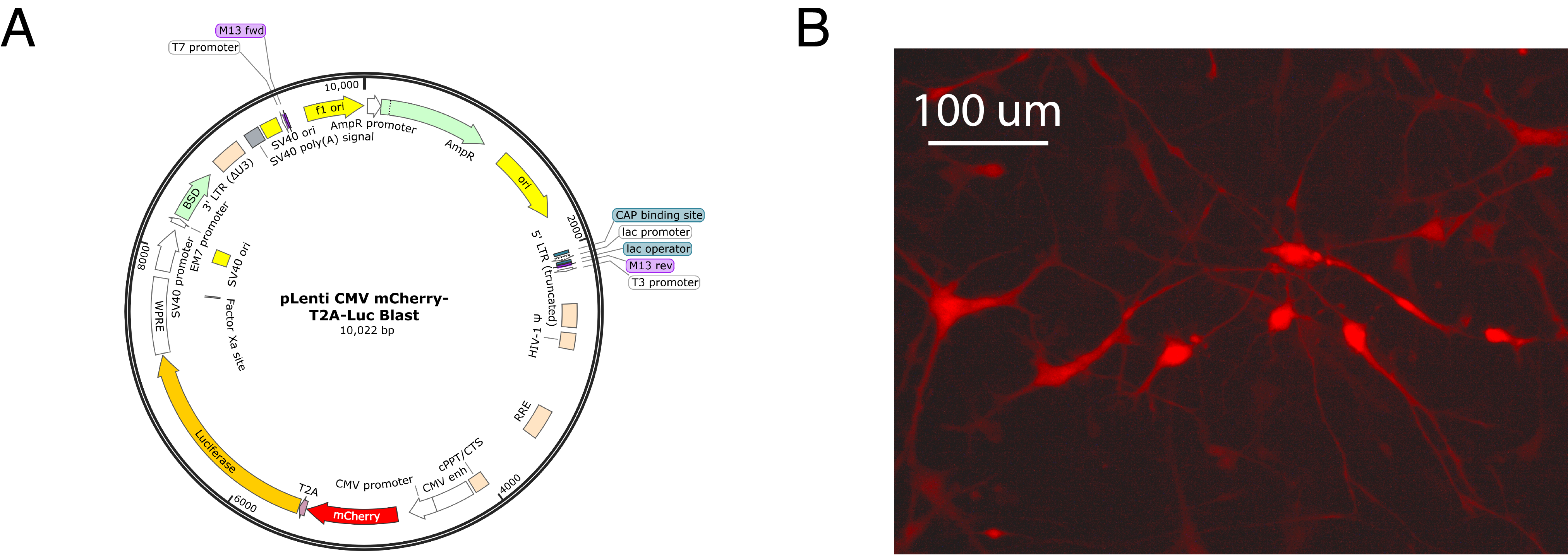

Supplement: Supplementary Figure 1 [file NIHMS1675447-supplement-Supplementary_Figure_1.png]
